# The PhanSST global database of Phanerozoic sea surface temperature proxy data

**DOI:** 10.1038/s41597-022-01826-0

**Published:** 2022-12-06

**Authors:** Emily J. Judd, Jessica E. Tierney, Brian T. Huber, Scott L. Wing, Daniel J. Lunt, Heather L. Ford, Gordon N. Inglis, Erin L. McClymont, Charlotte L. O’Brien, Ronnakrit Rattanasriampaipong, Weimin Si, Matthew L. Staitis, Kaustubh Thirumalai, Eleni Anagnostou, Marlow Julius Cramwinckel, Robin R. Dawson, David Evans, William R. Gray, Ethan L. Grossman, Michael J. Henehan, Brittany N. Hupp, Kenneth G. MacLeod, Lauren K. O’Connor, Maria Luisa Sánchez Montes, Haijun Song, Yi Ge Zhang

**Affiliations:** 1 Smithsonian National Museum of Natural History, Department of Paleobiology, Washington, DC 20560 USA; 2 University of Arizona, Department of Geosciences, Tuscon, AZ 85721 USA; 3 University of Bristol, School of Geographical Sciences, Bristol, BS8 1SS UK; 4 Queen Mary University of London, School of Geography, London, E1 4NS UK; 5 University of Southampton, School of Ocean and Earth Science, National Oceanography Centre Southampton, Southampton, SO14 3ZH UK; 6 Durham University, Department of Geography, Durham, DH1 3LE UK; 7 University College London, Department of Geography, London, WC1E 6BT UK; 8 Texas A&M University, Department of Oceanography, College Station, TX 77843 USA; 9 Brown University, Department of Earth, Environmental and Planetary Sciences, Providence, RI 02912 USA; 10 University of Edinburgh, School of Geosciences, Edinburgh, EH8 9XP UK; 11 GEOMAR Helmholtz Centre for Ocean Research Kiel, 24148 Kiel, Germany; 12 Utrecht University, Department of Earth Sciences, Utrecht, 3584 CB The Netherlands; 13 University of Massachusetts Amherst, Department of Geosciences, Amherst, MA 01003 USA; 14 Goethe University Frankfurt, Institute of Geosciences, 60438 Frankfurt am Main, Germany; 15 Université Paris-Saclay, Laboratoire des Sciences du Climat et de l’Environnement, Gif-sur-Yvette, France; 16 Texas A&M University, Department of Geology and Geophysics, College Station, TX 77843 USA; 17 GFZ German Research Centre for Geosciences, Section 3.3 Earth Surface Geochemistry, 14473 Potsdam, Germany; 18 Oregon State University, College of Earth, Ocean and Atmospheric Sciences, Corvallis, OR 97331 USA; 19 University of Missouri, Department of Geological Sciences, Columbia, MO 65211 USA; 20 University of Manchester, Department of Earth and Environmental Sciences, Manchester, M13 9PL UK; 21 University of East Anglia, School of Environmental Sciences, Norwich, NR4 7TJ UK; 22 China University of Geosciences, State Key Laboratory of Biogeology and Environmental Geology, School of Earth Sciences, Wuhan, 430074 China

**Keywords:** Palaeoclimate, Palaeoceanography

## Abstract

Paleotemperature proxy data form the cornerstone of paleoclimate research and are integral to understanding the evolution of the Earth system across the Phanerozoic Eon. Here, we present PhanSST, a database containing over 150,000 data points from five proxy systems that can be used to estimate past sea surface temperature. The geochemical data have a near-global spatial distribution and temporally span most of the Phanerozoic. Each proxy value is associated with consistent and queryable metadata fields, including information about the location, age, and taxonomy of the organism from which the data derive. To promote transparency and reproducibility, we include all available published data, regardless of interpreted preservation state or vital effects. However, we also provide expert-assigned diagenetic assessments, ecological and environmental flags, and other proxy-specific fields, which facilitate informed and responsible reuse of the database. The data are quality control checked and the foraminiferal taxonomy has been updated. PhanSST will serve as a valuable resource to the paleoclimate community and has myriad applications, including evolutionary, geochemical, diagenetic, and proxy calibration studies.

## Abstract (summary)

**Table:** 

Measurement(s)	sea surface temperature proxy values
Technology Type(s)	various
Sample Characteristic - Organism	Planktonic Foraminifera • Brachiopoda • Mollusca • Conodonta • Thaumarchaeota • Haptophyte
Sample Characteristic - Environment	marine biome
Sample Characteristic - Location	global

## Background & Summary

Geochemical proxy temperature data from ancient oceans are a key component of paleoclimate research^1–3^. High-resolution paleotemperature data from a single site can identify both long-term^4,5^ and orbital-scale^6,7^ climate variability, while multi-site comparisons from a given time slice provide insight into the spatial patterns of past climate change^8–12^. Additionally, such data are essential for validating Earth system models (ESMs)^13–16^ and provide critical context for first-order temporal trends in other aspects of the Earth system, including evolution^17,18^, geochemical cycling^19,20^, and tectonics^21,22^. While single-proxy data sets that span the Phanerozoic^23–25^ and multi-proxy compilations for select time slices^8–10,15^ exist, a comprehensive, multi-proxy database of temperature data spanning the Phanerozoic has yet to be published.

PhanSST, a database of sea surface temperature (SST) proxy data spanning the Phanerozoic Eon, seeks to fill this gap. The compilation currently contains 150,691 discrete proxy values, which can be used to estimate past SST. These data come from five different proxies, amassed from 660 references, and represent more than 1,600 unique sampling locations. Each proxy value is associated with a suite of consistent and queryable metadata fields, and the database is available in a machine-readable format. To the best of our knowledge, this is the largest compilation of Phanerozoic paleotemperature proxy data to date. Our intention is to make PhanSST a living database, growing, improving, and evolving through time. Accordingly, we encourage the paleoclimate community to contribute their published data to the compilation going forward.

In addition to the initial purpose of the compilation outlined below, PhanSST is an invaluable resource to the paleoclimate community and can be used for a wide range of additional applications, including evolutionary and geochemical studies. The spatial and temporal reach of PhanSST facilitates paleoclimatic syntheses, including studies investigating climate sensitivity, the biotic and geochemical impacts of climate change, and the mechanisms driving large-scale global climate. The data are presented in their native proxy units, which permits flexibility in the choice of calibration model and additionally enables the compilation to be used in proxy calibration studies. Further, PhanSST includes data from known and suspected diagenetically altered material, with both expert-assigned binary diagenesis flags and additional metadata fields, allowing the database to be used in research investigating spatial and temporal trends in preservation.

Version 0.0.1 of PhanSST is presented in a tabular (csv) format. A static copy of Version 0.0.1 is archived in the NOAA-NCEI Paleoclimatology Database (https://www.ncei.noaa.gov/access/paleo-search/study/36813)^26^, and dynamic versions of the most recent release can be found on Zenodo (10.5281/zenodo.7049233)^27^ and at the PhanSST website (https://www.paleo-temperature.org). In the following sections, we provide information regarding the data sources and selection criteria, review the definitions and decision-making behind the metadata fields associated with each proxy measurement, and outline the quality control process. We explore the broad spatial and temporal trends of the compilation, discuss future usage and limitations of the data set, and address the ongoing goals for the database to ensure that it remains a valuable asset to the paleoclimate community.

## Methods

### What is the primary purpose of the compilation?

The PhanSST database is a part of the broader PhanTASTIC (Phanerozoic Technique Averaged Surface Temperature Integrated Curve) initiative^28^, which aims to produce an internally consistent and statistically robust record of Earth’s global mean surface temperature over the last 539 million years. Ultimately, these data will be integrated with ESMs^29^ using data assimilation^10,30^ to reconstruct relevant global climate fields and calculate the global mean surface temperature at the geochronologic age (i.e., stage) level across the Phanerozoic. Requisite to this goal is a compendium of paleotemperature proxy data.

PhanSST is a community-wide, collaborative effort. Each of the authors of this data descriptor contributed their time and expertise, entering data and quality control checking the records. Despite the more focused primary purpose of the compilation, we want to ensure that PhanSST will serve as a community-wide resource. The curated metadata fields, therefore, reflect a desire to maximize potential reuse of the database.

### Why geochemical SST proxy data?

We focused on compiling geochemical SST proxy data for several reasons. First, the ocean comprises approximately 70% of the Earth’s surface, and SST proxies provide better long-term temporal coverage and generally more precise age control than terrestrial records due to more continuous deposition in marine environments. Second, the proxy types in PhanSST can be readily converted from proxy values into temperature units using established calibration models that propagate errors and account for seasonal biases^31–36^. Third, terrestrial air temperature proxies are a function of elevation and lapse rate, making interpretations of terrestrial data dependent upon paleogeographic and paleoaltimetric assumptions that are poorly constrained in deep time. SST proxies are still subject to assumptions, such as seawater chemistry, seasonality and depth of production, and temporal uniformity of proxy systems^34,35,37^, and interpretations can be sensitive to the influence of plate movement or shifts in surface current positions^38,39^. However, the comparative richness of the marine record generally makes identifying anomalous sites or entries more straightforward. While beyond the scope of our current efforts, we hope that in the near future a parallel compilation of terrestrial temperature proxies will be developed.

### Which SST proxy data?

PhanSST included data from five SST proxies: oxygen isotopes of macro- and microfossil carbonate (*δ*^18^O_*carbonate*_), oxygen isotopes of conodont phosphate (*δ*^18^O_*phosphate*_), magnesium to calcium ratios (Mg/Ca) of planktonic foraminifera, the tetraether index of 86 carbons (TEX_86_), and the alkenone unsaturation ratio (${{\rm{U}}}_{37}^{K{\prime} }$). While we did also collect marine carbonate clumped isotope data (Δ_47_), those data are not included in the current release (Version 0.0.1) of PhanSST. Although laboratory-specific Δ_47_-temperature calibrations are robust, interlaboratory differences in the way Δ_47_ data have been corrected raise concerns over the compatibility of the isotope values themselves^40^. Since PhanSST presents proxy values, rather than paleotemperature estimates, we opted to omit the Δ_47_ data for now. With that said, a newly proposed reference frame^40^ offers a promising way forward and we hope to include these data in the near future.

All of the proxy data included in the database have been previously published in peer-reviewed journal articles, theses and dissertations, book chapters, or public repositories (e.g., PANGAEA)^4–7,24,41–695^; the database does not include any unpublished data. The proxy values themselves, dominantly derive from either data tables contained within the original journal article, supplements, or public repositories. In general, data locked in PDF tables were extracted using Tabula (https://tabula.technology). Some dark data and missing metadata were obtained through personal communication with the original authors and, in very rare instances, data were digitized from figures. Details regarding the source of dark data can be found in the Quality Control logs (see *Compilation quality-control* for log availability).

PhanSST expands upon several existing compilations. The majority of oxygen isotope values from carbonate macrofossils come from Grossman and Joachimski^23^ and the initial references of phosphate oxygen isotope values mostly derive from Grossman and Joachimski^23^ and Song *et al*.^25^. Likewise, many of the Cretaceous data were originally collated by O’Brien *et al*.^696^, Paleogene data by Hollis *et al*.^8^ and Evans *et al*.^697^, and Pliocene data by McClymont *et al*.^9^. To the extent possible, data sourced from these compilations were cross-checked with their initial publications to ensure completeness and avoid propagating any unintentional errors, and missing data or applicable metadata fields were filled in. The remaining data in PhanSST were scoured from the literature by the authors, who worked in teams based on their expertise with specific proxies and geologic time intervals. We used keyword queries in Google Scholar to identify missing references, and efforts were made to target literature from data-poor regions (e.g., South America) and time intervals (e.g., Silurian). While (quasi-)automated data discovery and entry methods show promise as a means of maximizing database completion and minimizing bias^698^, given the broad nature of data sources and formats, such an approach was not tenable here.

Selection criteria varied slightly by proxy and time interval. Thanks to the initiatives of the International Ocean Discovery Program (IODP) and its predecessors, late Mesozoic and Cenozoic SST data are plentiful and data derived from cores often consist of high-resolution time series. In this case, we focused on compiling time series spanning at least one million years, recognizing that Quaternary data, for example, are compiled in more detail in other works^699,700^. Exceptions to this rule were made for data from undersampled regions (e.g., the Southern Ocean) or time periods (e.g., the Paleocene). In contrast to the high data density of the Cenozoic, data from the early Mesozoic and Paleozoic are comparatively scarce. Several proxies, such as ${{\rm{U}}}_{37}^{K{\prime} }$, TEX_86_, and foraminiferal-based records are limited to more recent times, and almost all ocean crust older than ~180 Ma has been recycled through subduction^701^, removing a key sampling environment. As such, data availability is restricted by outcrop exposure, subject to more frequent and larger unconformities, and limited to fossiliferous horizons. Compared to core data, these records generally contain fewer measurements per site, lack a continuous time series, and have less precise age control or report relative rather than numeric ages. Therefore, we incorporated all available Paleozoic and Mesozoic proxy data, regardless of record duration. We did, however, require that all sites provide some level of age control; data that could not be assigned a relative age at the stage level were excluded.

### Which metadata fields are included and why?

The metadata fields included in PhanSST reflect a balance between the scope of the PhanTASTIC project and our intention to maximize the reuse potential of the database. Metadata fields were carefully curated to facilitate future updates to age models and proxy-, species- or methodological-specific corrections. For example, core information, sampling depth, and biostratigraphic information are included, permitting age model updates. On the other hand, we do not list age uncertainties because (1) age uncertainties are not consistently reported in the source publications, (2) it was not tractable to update all age models to a consistent timescale, and (3) the PhanTASTIC project focuses on temperature evolution at the stage level. Since we strive to make PhanSST a living database, we anticipate that updated age models, with uncertainties, may be added at a later date with assistance from the paleoclimate community.

To ensure a comprehensive and methodologically traceable compilation, we also opted to include diagenetically altered samples. Some samples can confidently be characterized as altered; however, diagenetic processes are gradual and criteria for what constitutes diagenetically altered material can be subjective and may change as new insights into diagenetic processes are gained. Consequently, rather than excluding such data, we have: (1) applied an expert-assigned binary diagenesis flag to make it clear which samples have been previously suggested or interpreted as altered, and (2) included relevant supplementary fields, such as elemental concentrations^702^ and conodont color alteration index (CAI) values^703^, allowing users to impose their own informed diagenetic assessments. This approach ensures that all data are available for future studies that may wish to adopt different alteration criteria or explore the spatial and temporal patterns of diagenesis.

### Which metadata fields are excluded and why?

The metadata fields currently provided have been carefully selected to help end users make informed decisions of which data to include in analyses and how best to correct them. However, two key metadata fields are omitted from PhanSST: paleotemperature estimates and paleocoordinates. Though we acknowledge that these fields are of particular interest, this was done deliberately to promote responsible and intentional reuse of the database.

Estimating SSTs from the proxy values involves applying and inverting a statistical model of how each proxy responds to environmental parameters; it is therefore a derived or modeled quantity subject to user-based decisions about how to treat each proxy system. In order to estimate SST for each of the more than 150,000 data points, we would need to make executive decisions regarding (1) which calibration to use for each proxy system, (2) what assumptions to use for non-thermal predictor variables (such as seawater chemistry), and (3) which (if any) taxon-specific or analytical corrections to apply. For any given proxy, there are a variety of proxy system models (PSMs) with which to estimate SST. For example, within the TEX_86_ proxy system, SSTs can be calculated using the ${{\rm{TEX}}}_{86}^{H}$^704^, TEX_86-linear_^696^, or BAYSPAR^31^ calibrations, among others, and exploration into region- and time-specific modifications to these calibrations is ongoing (e.g., Steinig *et al*.^574^). Holding all else constant, the difference in inferred temperatures across these calibrations can exceed 5 °C, and there is no consensus among the paleoclimate community on the most appropriate calibration(s) to use^8,705^. In fact, the choice of which calibration is most appropriate can vary based on the time interval and temporal resolution of the study, the taxon from which the data derive, the geographic breadth of the data, and the types of questions being investigated. Similarly, in order to calculate SST, many of these proxy systems require assumptions about non-thermal predictor variables (e.g., *δ*^18^O_*seawater*_). Relating the proxy values to SST would therefore require decision making about these seawater chemistry values in the geologic past, which are largely unconstrained. Given the volume of data contained within PhanSST, these assumptions would need to be based on broad, first-order principles (e.g., a standardized *δ*^18^O_*seawater*_ of −1‰ for ice-free times and 0‰ value during times of glaciation), but such simplifications are incorrect (e.g., we observe a >5‰ spatial spread in values in the modern ocean^706^, a nuance that is wholly overlooked by assuming a single “ice-free” or “glaciated” value). Though there are better ways of estimating these values (e.g., extracting the local *δ*^18^O_*seawater*_ values from an isotope-enabled ESM), such methods cannot be consistently applied across the entire database. And, in fact, the necessary resolution of these assumptions changes based on the scope of the study. Studies, for example, investigating Phanerozoic trends in oxygen isotopes may wish to apply a single calibration to all data points and use simplified assumptions for consistency, whereas studies investigating a specific time slice (e.g., the Cretaceous), may benefit from taking a more intricate approach (e.g., using taxa-specific PSMs and drawing *δ*^18^O_*seawater*_ values from ESMs). It is therefore not possible for us to calculate SSTs for each data point while applying a traceable, scientifically sound, and ubiquitously appropriate methodology. Moreover, it is important that those who wish to calculate SSTs from the proxy data within PhanSST be fully aware of the decision making (and uncertainty) associated with the various PSMs and assumptions.

Likewise, we do not include paleogeographic information, which is also a derived quantity based on geophysical models. Here, we plot data using the plate rotation model of Scotese and Wright (2018)^707^ for illustrative purposes, since it is one of the few rotations that extends back through the Phanerozoic. However, there are a variety of different plate rotation models available, particularly in more recent time intervals, and the projected paleocoordinates can vary significantly based on the rotation model used^38^. As with the SST estimates, the choice of which paleorotation is most appropriate to use is dependent upon the application of the data and the temporal resolution of the study. If, for example, a user is plotting data on existing ESM output, then they will want to use the same rotation that was used in the simulation so that the paleolongitudes (which are largely unconstrained) align. Alternatively, if they are testing a hypothesis about the timing or influence of a specific gateway opening, they may wish to use several different rotations and compare the results. Further, when aligning data in deep time, the time scale of the study matters, and the choice of time interval with which to align the data will again be dependent on the scope of the investigation. Time slice studies may wish to rotate all data to the same time frame (e.g., 56 Ma) despite the fact that the data may span a few million years in either direction. Other studies looking at changes in ocean dynamics from the same site across a 5-million-year window may wish to rotate each discrete data point to their precise numeric age. Given the variety of methods for estimating both SST and paleocoordinates, and the inherent complexity and uncertainty behind those singular discrete values, we believe that it is important for end users to go through the decision making process themselves to ensure that the values are best tailored toward their respective application. We do, however, recognize that users who wish to calculate SSTs or paleocoordinates may benefit from guidance. We offer some broad recommendations on how to select and apply temperature calibrations and rotation models below in the *Applying the database in deep time* section.

## Data Records

In addition to the proxy value itself, each entry is associated with an array of relevant metadata fields, which vary depending on the nature of the record and the proxy. In total, there are more than 40 metadata fields that can broadly be grouped into six categories: (1) sample site and other identifying information, (2) age information, (3) proxy information, (4) taxonomic and environmental information, (5) proxy-specific information, and (6) reference metadata. Basic descriptions of these fields can be found in Table 1 and specifics on how and why each field is assigned are provided below.

**Table 1:** Field names and descriptions.

Field name	When applicable	Description of field (units)
*Sample ID and location fields*		
SampleID	All data	Unique sample identification code, as originally published
SiteName	All data	Name of the drill core site or section
SiteHole	Drill core data	The alphabetic hole specifier
MBSF	Drill core data	Depth below the sea floor (m)
MCD	Drill core data	The mean composite depth (m)
SampleDepth	Outcrop data	Stratigraphic height or depth (m)
Formation	Outcrop data	Geologic formation name
Country	All data	The country or ocean of the data collection site
ContinentOcean	All data	The continent or ocean basin of the data collection site
ModLat	All data	Modern latitude of collection site rounded to two decimals; negative values indicate the Southern Hemisphere (decimal degrees)
ModLon	All data	Modern longitude of the collection site rounded to two decimals; negative values indicate the Western Hemisphere (decimal degrees)
*Age fields*		
Age	All data	Age, in reference to GTS2020 [710] (Ma)
Period	All data	The geologic period
Stage	All data	The geologic stage (i.e., geochronologic age)
StagePosition	All data (barring those only assigned to a stage)	Further specification of relative age (Early, Middle, or Late)
Biozone	Outcrop data	Conodont, graptolite, and/or ammonite biozone
AgeFlag	All data	Flag indicating if relative age fields were autofilled from numeric age values (0), age values were autofilled from relative age fields (1), or both relative and numeric age values were provided (2)
*Proxy fields*		
ProxyValue	All data	Reported proxy value (native proxy units)
ProxyType	All data	Reference to the proxy type; see Table 2
ValueType	All data	Reference to the averaging of the data (see text)
DiagenesisFlag	*δ*^18^O and Mg/Ca data	Binary expert-assigned flag indicating good (0) or questionable (1) preservation
*Taxonomic, environmental, and ecological fields*		
Taxon1	All data	First-order taxonomic classification (see Table 3)
Taxon2	*δ*^18^O data	Second-order (class) classification of mollusks (see Table 3)
Taxon3	All *δ*^18^O and Mg/Ca data	Third-order (genus or species) classification (see Table 3)
Environment	Outcrop data	Depositional environment (e.g., mid-shelf, epeiric)
Ecology	All *δ*^18^O and Mg/Ca data	Ecological preference of the sampled taxon (e.g., surface, benthic)
*Proxy-specific fields*		
AnalyticalTechnique	All *δ*^18^O data	The analytical technique used to obtain the data (IRMS vs SIMS)
CL	Macrofossil *δ*^18^O_*carbonate*_ data	Cathodoluminescence microscopy assessment
Elemental Suite	Macrofossil *δ*^18^O_carbonate_ data	All reported elemental concentrations (i.e., Fe, Mn, Mg, Sr, Ca) or ratios (i.e., Sr/Ca, Mg/Ca, Mn/Sr)
NBS120c	*δ*^18^O_phosphate_ data	The NBS120c standard value used to correct the data (‰)
Durango	*δ*^18^O_phosphate_ data	The Durango standard value used to correct SIMS data (‰)
MaximumCAI	*δ*^18^O_phosphate_ data	The maximum reported conodont color alteration index value for that sample or horizon
ModWaterDepth	Mg/Ca data	The modern water depth of the sampling site
CleaningMethod	Mg/Ca data	A binary flag to indicate either oxidative-only cleaning (0) or inclusion of a reductive cleaning step (1)
Fractional abundances	TEX_86_ data	Fields to indicate the fractional abundances of the GDGTs
Index values	TEX_86_ data	Branched and isoprenoid tetraether (BIT), methane (MI), and delta ring (dRI) index values
*Reference metadata*		
LeadAuthor	All data	The last name of the first author of the original publication
Year	All data	The year of the original publication
PublicationDOI	All data	The DOI of the original publication
DataDOI	All data	The DOI of the online repository hosting the data

For clarity and consistency while reading this data descriptor, ***entries*** refer to each discrete proxy value and its associated metadata (i.e., a row), ***fields*** refer to the metadata collected for each entry (i.e., a column), and ***sampling sites*** refer to the unique geographic coordinates of entries, independent of the number entries from that location. Unique sampling sites can be parsed temporally (e.g., by stage, period, 2.5 myr bins, etc.), by proxy type, or a combination of the two.

### Sample ID and location fields

The first field is the *SampleID*, which reflects the unique (often alphanumeric) identification code associated with each entry, as originally published. Currently, the *SampleID* field only applies to samples collected in outcrop; however, in the future, we plan to also include the unique drill core sample IDs. The remaining sample site fields provide information on the geographic and stratigraphic position of each entry. Data that come from drill cores, such as those from IODP, list the *SiteName*, referencing the expedition site, and also include sampling depth information (i.e., *SiteHole*, *MBSF*, and/or *MCD*) to allow age models to be updated in the future. Data collected in outcrop similarly include a *SiteName* and *SampleDepth*, as well as the geologic *Formation* from which the data were collected, when available.

Each entry is also associated with the modern coordinates of the sampling site (*ModLat* and *ModLon*) and tags indicating the *ContinentOcean* and *Country* of collection for easy filtering. For consistency, the coordinates of sample sites were rounded to two decimal degrees. In some cases (predominantly Paleozoic data collected in outcrop and published in older journal articles) precise coordinates were not provided^423,483,501^. In these instances, locations were estimated using context from the original publication, outcrop information from Macrostrat^708^, and occurrence data from the Paleobiology Database^709^.

### Age fields

Six age fields provide information related to the age of each sample, including numeric *Age* (in Ma) as well as the *Period* and *Stage*, which provide relative age information. We use the chronostratigraphic term “stage” as a synonym for the geochronologic “age” to mitigate confusion between numeric ages and geochronologic ages. Biostratigraphic information (e.g., conodont zone), when provided, is retained in the *Biozone* field, permitting future age model updates for data collected in outcrop with each new iteration of the Geologic Time Scale (GTS). We did not divide data from the Holocene Epoch into their respective stages; all data younger than 0.0117 Ma were assigned to the “Holocene” in the *Stage* field. Likewise, all data from the unnamed stage of the Pleistocene were assigned to the “Upper Pleistocene” and all data from the Pridoli Epoch are assigned to the “Pridoli”.

Given the broad nature of this compilation and the variety of time scales and proxies incorporated, constraining entry ages in a consistent and traceable way required a multifaceted approach. Data coming from IODP cores, for example, often have higher precision age models tied to sampling depth. In contrast, data from outcrops are frequently more challenging to date precisely and quantitatively. Thus, ages were assigned in one of three ways: by entering a numeric age and auto-filling the relative age information, by entering relative age information and auto-filling the numeric age information, or by retaining both the manually entered numeric and relative age information. The *AgeFlag* field identifies which approach was taken (indicated by a 0, 1, or 2, respectively).

#### Numeric age assignments with auto-filled relative ages

If the original paper provided a precise numeric age, the period and stage fields were automatically filled in using age boundaries from GTS 2020^710^. We made efforts to use recent drill core age models, but given the size of the data compilation, updating all of the age models was not feasible. When available, we compiled sampling depth information (i.e., *SiteHole*, *MBSF*, and/or *MCD*) so that age models may be updated in the future. If the data came from an existing compilation, we deferred to their reported age unless a more recent age model was readily available. Data with precise numeric ages are denoted by a zero (0) in the *AgeFlag* field.

#### Relative age assignments with auto-filled numeric age

Some data can only be relatively dated. In the absence of precise numeric ages, the *Stage* field was entered manually. Stage duration is highly variable, with a median length of 4.65 myr. In general, stage duration scales with availability of material and scientific interest in the time interval, with a maximum duration of 21.56 myr (Norian) and a minimum of 11.7 kyr (Holocene, though not a defined stage as discussed above).

The stage assignments are largely based on the divisions reported in the original publication, though some data points have been updated based on the biostratigraphic information contained in the *Biozone* field based on the divisions in GTS 2020^710^. Relative ages were further qualified using the *StagePosition* field (i.e., early, middle, or late), if such information was available or could be realistically constrained using the biozonations. A numeric age was then estimated based on the stage boundaries of GTS 2020^710^. Entries constrained only at the stage level were assigned numeric ages based on the arithmetic mean of the upper and lower stage boundaries. Numeric ages of entries tied to a specific stage position (early, middle, or late) were estimated by dividing the stage into three equal time slices and assigning a numeric age based on the midpoint of that entry’s respective third. Data with numeric age assignments extrapolated from relative age information are denoted by a one (1) in the *AgeFlag* field.

Note that assigning ages in this manner means that high(er)-resolution relative time series information is not retained in the auto-filled numeric ages. For example, in some original publications, authors constructed their own relative age model and assigned sequential relative ages to stratigraphically successive data points. However, it was not feasible to convert these relative time series into numeric ages using a consistent and traceable methodology. For the purposes of the PhanTASTIC project, stage-level temporal resolution is sufficient. Regardless, stratigraphic position, when available, was recorded in the *MBSF*, *MCD*, and/or *SampleDepth* fields, which allows users to construct relative time series if desired.

### Proxy fields

The four proxy fields consist of the proxy value, the proxy type, value type, and preservation state. Table 2 provides a list of all *ProxyType* options. *ValueType* options include: (1) ‘im’, indicating the proxy value represents an individual measurement from an individual specimen, (2) ‘ia’, indicating the proxy value reflects the average of replicate measurements from a single individual specimen, and (3) ‘pa’, indicating the measurement reflects a population average from multiple specimens. All TEX_86_ and ${{\rm{U}}}_{37}^{K{\prime} }$ entries, by definition, are listed as ‘pa’.

**Table 2:** Proxy types.

Field value	Proxy type	Units
d18a	*δ*^18^O of aragonite	‰; VPDB
d18c	*δ*^18^O of calcite	‰; VPDB
d18p	*δ*^18^O of phosphate	‰; VSMOW
mg	Mg/Ca ratio	mmol/mol
tex	TEX_86_	unitless ratio
uk	${{\rm{U}}}_{37}^{K{\prime} }$	unitless ratio

The *DiagenesisFlag* field is a binary flag specifying preservation state. We generally deferred to the expert opinion of the authors responsible for entering and/or quality-control checking the data and were as conservative as possible, flagging both known and suspected alteration. Both *δ*^18^O and Mg/Ca foraminiferal data were flagged based on the preservation of the tests themselves (e.g., glassy vs. frosty)^466^ rather than an assessment of the fidelity of the proxy value. Unlike foraminifera, there is currently no method for assessing the preservational state of the macrofossil *δ*^18^O_*carbonate*_ and *δ*^18^O_*phosphate*_ that is consistently applied across the literature. The diagenetic flags provided here, therefore, generally reflect the interpretation of the original authors. We also flagged any *δ*^18^O_*carbonate*_ entries lower than −10‰ as these values would yield unrealistically warm SSTs, suggesting that either diagenesis or non-marine seawater compositions affected the measurement. We appreciate that diagenesis is a spectrum and applying a subjective binary flag both overlooks the nuance of the processes involved and is inherently not reproducible. To that end, we have also provided additional diagenetic fields specific to *δ*^18^O_*carbonate*_ and *δ*^18^O_*phosphate*_ data, such as trace and major element ratios and concentrations of macrofossils, cathodoluminescence assessments, and maximum CAI of conodonts. End users can define their own diagenetic thresholds (e.g., Mn/Sr <0.5) and use these fields to consistently filter or flag suspect data.

We have not indicated the preservation state of TEX_86_ nor ${{\rm{U}}}_{37}^{K{\prime} }$ entries because although methods for assessing preservation and thermal maturity exist^8,711^, they are not consistently reported in the literature. Nevertheless, as with the oxygen isotope data, we have included some proxy-specific fields to help filter TEX_86_ data for non-thermal influences (see below).

### Taxonomic and environmental fields

The taxonomic and environmental fields provide information about the organism from which the proxy data derive (Table 3), as well as the depositional environment and ecology of the sampled taxon. The *Taxon1* field refers to the first-order taxonomic affiliation of the organism, primarily classified at the phylum level. The *Taxon2* field, applicable only to mollusk *δ*^18^O_*carbonate*_ data, further specifies the class, and the *Taxon3* field specifies the binomial species name of the sampled organism, when available. These fields help characterize the paleoenvironment from which the data come, permit the compilation to be filtered by taxon, and contain information pertinent to species-specific calibrations^34,35^.

**Table 3:** Taxonomic specifiers.

Taxon1	Taxon2	Taxon3	Description
br		Binomial species name	Brachiopod
m			Mollusk
	bi	Binomial species name	Bivalve
	ce	Binomial species name	Cephalopod
	ga	Binomial species name	Gastropod
	ot	Binomial species name	Other
co		Binomial species name	Conodonts
ha			Haptophyte
pf		Binomial species name	Planktonic foraminifera
th			Thaumarchaeota

While most of the entries in PhanSST are reported as representing SST, in reality very few of the taxa from which the data derive genuinely lived at the sea surface. Some PSMs, such as BAYSPAR^31^ for TEX_86_ and BAYSPLINE^33^ for ${{\rm{U}}}_{37}^{K{\prime} }$, are based on calibrations between core tops and modern SST, partly (but not completely, since organisms might migrate vertically through time) alleviating this limitation. However, it can be difficult to interpret data that come from (1) proxies that do not have modern core top calibrations, (2) extinct taxa, or (3) intervals with substantially different environmental conditions than today that may have promoted vertical migration within the water column. When available and applicable, we have therefore included metadata pertaining to the depositional environment of the source material (e.g., marginal, mid-shelf, slope) in the *Environment* field and the lifestyle or depth habitat of the organism in the *Ecology* field. The ecology of carbonate macrofossils and conodonts are classified as either benthic, nektic, planktic, or some combination therein, while planktic foraminifera are classified as either surface/mixed layer or (sub-)thermocline. These fields allow users to easily filter or index entries within the compilation to fit their needs. For example, depending on the scope of the study, it may be germane to filter out benthic brachiopods deposited in slope environments while retaining brachiopods from inner shelf environments, as they are more likely to record temperatures closer to sea surface values. Alternatively, comparing data from surface- and thermocline-dwelling species of foraminifera with benthic bivalve data from the same site could elucidate vertical temperature gradients of ancient oceans.

### Proxy-specific fields

Certain proxy-specific fields were added to the compilation to permit data corrections, facilitate the implementation of PSMs, aid in diagenetic assessments, and improve the overall utility of the database. For traceability purposes (i.e., to ensure that our records reflect the original data tables) we have not corrected any of the proxy values, but we do provide the information required to enable a correction. For example, the *δ*^18^O data from both carbonates and phosphates include information about the *AnalyticalTechnique* (i.e., IRMS or SIMS), as evidence suggests there may be methodological offsets in the isotope values^148,712^. The NBS-120c value used to standardize the *δ*^18^O_*phosphate*_ data is reported in the *NBS120c* field. The consensus value of 21.7‰ is now generally applied, but the value of the standard varies significantly in older literature. Additionally, in the case of SIMS data, we also report the *Durango* standard value. Reporting the “uncorrected” published values, while also specifying the method, permits end-user autonomy of how to treat data derived by different means.

To assist in assessments of sample preservation, we have included available trace, minor, and major element concentrations and ratios associated with *δ*^18^O_*carbonate*_ macrofossil data (e.g., Fe, Mn, Mg, Sr, Sr/Ca, Mg/Ca). Diagenetic processes yield predictable directional changes to these concentrations and ratios; by comparing fossil values to modern taxon- or site-specific ranges, informed threshold values can thus be used to further assess the preservation state of samples^713,714^. For the same reason, when reported, we have also included categorical diagenetic assessments using cathodoluminescence microscopy^702^ (*CL*; L, luminescent; SL, slightly luminescent; NL, non-luminescent).

When available, *δ*^18^O_*phosphate*_ entries are associated with the maximum reported conodont color alteration index (*MaximumCAI*) value^703^ for that sample or horizon. Phosphate is generally considered to be more resistant to diagenetic alteration than carbonate, but there remains some debate as to when data from conodont elements should be considered altered^148,312^. Inclusion of this field allows users to impose their own CAI threshold criteria for assessing preservation or to analyze relationships between isotope values and CAI.

Additional fields are included for foraminiferal Mg/Ca data to provide information needed for using the Bayesian Mg/Ca forward model, BAYMAG^35^. Previous studies have demonstrated that Mg/Ca values are dependent upon both the bottom water calcite saturation state^715^ (Ω) and the cleaning method used prior to analysis^716^, and, as such, are included as predictor variables in BAYMAG. All Mg/Ca entries, therefore, include the water depth of the modern sampling site (*ModWaterDepth*), which can be used in tandem with the geographic information to inform paleodepth estimates (and thus constrain Ω), and a binary *CleaningMethod* field to indicate whether the cleaning omitted (0) or included (1) a reductive step.

TEX_86_ entries include the fractional abundances of the isoprenoidal glycerol dialkyl glycerol tetraethers (GDGTs), if available, following the recommendation of Hollis *et al*.^8^. Additionally, we have included the branched and isoprenoid tetraether index^717^ (BIT), the methane index^718^ (MI), and the delta ring index^719^ (ΔRI), which can all be used to assess the extent to which TEX_86_ values may be affected by non-thermal factors. These fields allow the end-user to screen data by the indices and threshold values of their choice.

### Reference metadata

The reference fields contain the publication metadata, including the lead (first) author, publication year, and the DOI to the publication where the data were originally reported (*PublicationDOI*). We also recognize that many of these data are available in online repositories (e.g., PANGAEA). While Version 0.0.1 of PhanSST only provides the *PublicationDOI*, in future releases we plan to also utilize the *DataDOI* to direct users to any online repository hosting the data. To aid in ease of machine readability, accents and special characters in the *LeadAuthor* and other applicable fields were removed. However, the full and unaltered database citations, including the article title and journal name, can be found in a supplementary Excel (.xlsx) file available on Zenodo and the PhanSST website.

## Technical Validation

### Compilation quality-control

**Fig. 1:**
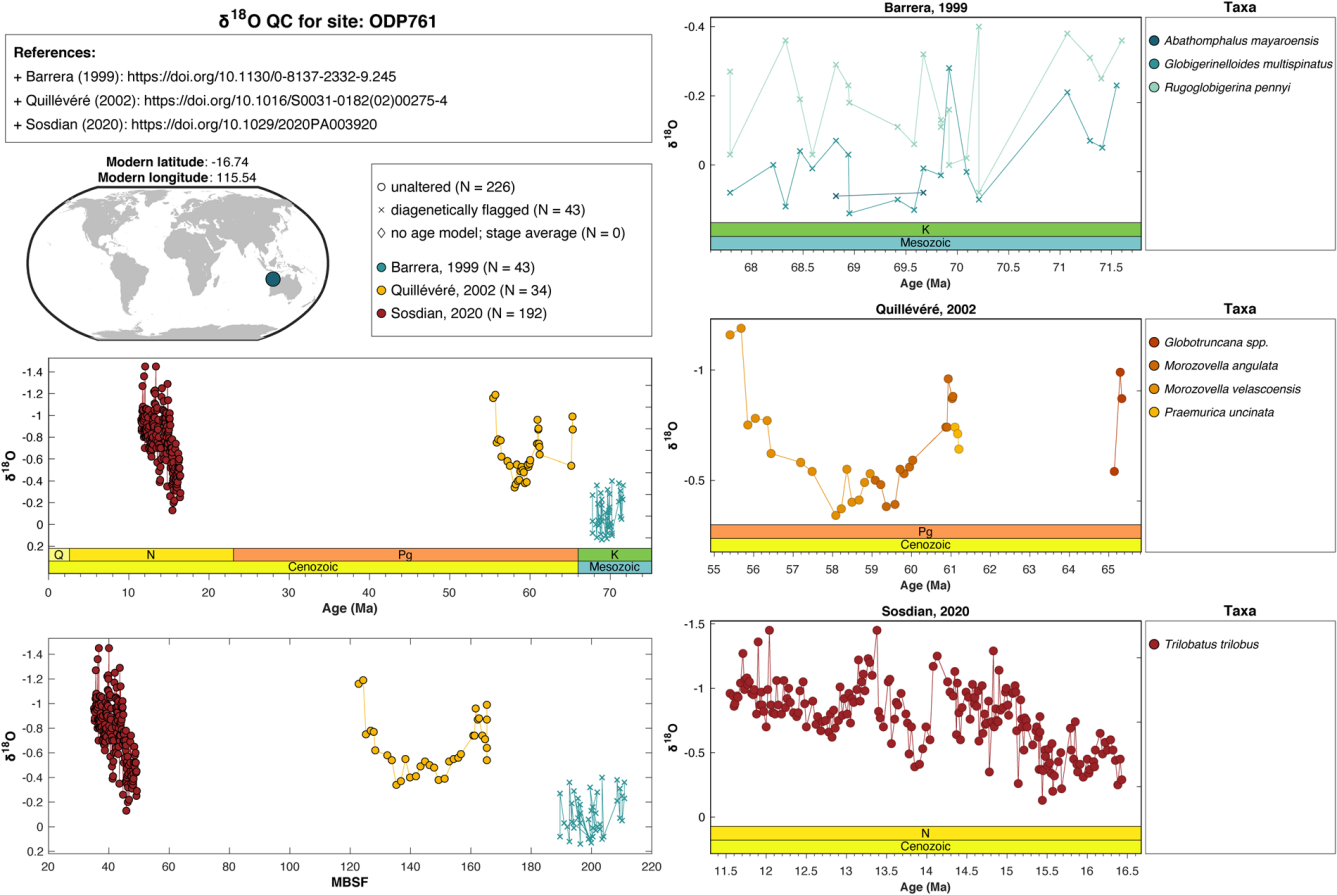
Example QC PDF for the foraminiferal *δ*^18^O_*carbonate*_ data from ODP Site 761.

**Fig. 2:**
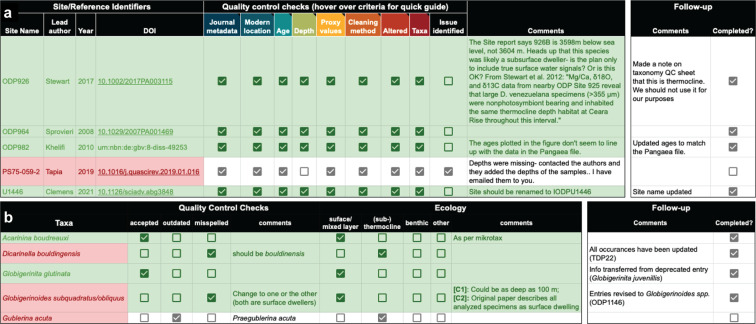
(**a**) Example QC Google spreadsheet for foraminiferal Mg/Ca data, parsed by site and publication. The fidelity of the entered data was checked based on eight suites of standardized criteria (see Fig. 3). (**b**) Example of the foraminifera taxonomy QC Google spreadsheet. Foraminiferal species were checked separately for outdated or misspelled names and flagged by environment.

**Fig. 3:**
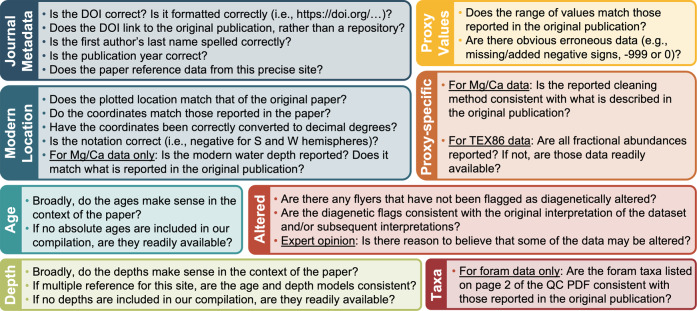
The QC criteria under which each site and reference was evaluated. Checking the boxes of the same color and heading in Fig. 2a confirms that all of the criteria for a given category have been met.

Data were quality-control (QC) checked by the authors of this data descriptor. For data deriving from drill cores, we generated a PDF file for each unique sampling site, parsed by the *SiteName* and *ProxyType* fields (Fig. 1). The QC PDFs included relevant metadata, such as the reference fields (*LeadAuthor, Year, DOI*) and the sample site coordinates. All data for a given site were plotted versus both time and core depth and colored by reference, with different symbols to indicate the *DiagenesisFlag* and *AgeFlag* assignments of each data point. QC PDFs of data derived from planktic foraminifera (*δ*^18^O_*carbonate*_ and Mg/Ca) contained plots colored by species. Using shared Google spreadsheets for each proxy, we verified that the information in each QC PDF matched the publication values, working in teams based on expertise (Fig. 2a). Each unique site, proxy, and reference was evaluated based on a standardized suite of criteria to ensure consistency throughout the process (Fig. 3). Those assisting in the QC process checked boxes to confirm the fidelity of the journal metadata, modern coordinates, age model, depth information, proxy value, preservation state, taxa, cleaning method (for Mg/Ca), and fractional abundances (for TEX_86_). We corrected any issues identified and maintained a detailed log of comments, providing a record of our QC process. Links to read-only versions of these QC logs are available on the PhanSST website and in the “Read Me” documentation on Zenodo.

Given the large number of foraminiferal species in the database and the potential for outdated taxonomy, we conducted a systematic QC of the listed species. Species names were checked for outdated taxonomic assignments and misspellings (Fig. 2b), using databases like Mikrotax. We added an expert-assigned *Ecology* field to help distinguish surface or mixed layer dwelling species from (sub-)thermocline species.

The macrofossil *δ*^18^O_*carbonate*_ and *δ*^18^O_*phosphate*_ data were QC checked separately. These data generally come from outcrops, with many studies reporting multiple sampling sites but only a few measurements at each site. These differences inhibited straightforward parsing of the carbonate macrofossil and phosphate records into site-level QC PDFs, so instead these data were inspected manually. Records were checked by comparing proxy values and metadata between existing compilations^23^ and original publications. As with the core data, a detailed log of the changes made was retained.

In addition to the manual quality-control checks described above, we also performed automated analyses for each field to identify any missing or erroneous metadata (available on GitHub; see *Code Availability*). For example, the *ModLat* and *ModLon* fields were screened to ensure that latitude and longitude values were in decimal degrees and fall between −90 to 90 and −180 to 180, respectively. Country names and sample coordinates were further crosschecked against publicly available country shapefiles data, to ensure accuracy and consistency in the assignment. *ContinentOcean* assignments were verified by indexing all categorical responses (e.g., NA, North America; SO, Southern Ocean) and mapping each data point tagged to each respective geographic region. Stage and period names were compared to the list of accepted names from GTS 2020^710^, and checked for agreement between numeric age, period, and stage. We also printed lists of the unique period, stage, proxy type, value type, and taxonomic names to ensure they each respectively conformed to our accepted convention. Lists of all unique values for each categorical field (e.g., *ProxyType, ValueType, AnalyticalMethod*) were printed to identify any inconsistencies, and every field was queried to identify missing values.

### General database statistics

Version 0.0.1 of PhanSST contains 150,691 entries, drawn from 660 different references, representing 1,643 unique sampling sites and 93 of the 100 Phanerozoic stages. Of these, 25,782 have been flagged as diagenetically altered, though this flag does not apply to the TEX_86_ or ${{\rm{U}}}_{37}^{K{\prime} }$ entries; there remain nearly 120,000 unflagged data points after applying a conservative screening of these latter proxies to ensure fidelity of their values (i.e., excluding TEX_86_ data whose BIT, MI, or ΔRI values are greater than 0.5 and ${{\rm{U}}}_{37}^{K{\prime} }$ data whose paleolatitude is greater than 70°N, since these data are considered compromised by alkenone production in sea ice^33,720,721^). Across the database, *δ*^18^O_*carbonate*_ data are the most common, constituting over half of all entries (Table 4). This prevalence reflects the fact that carbonate oxygen isotope data (1) are the oldest quantitative paleotemperature proxy, with its origins dating back to 1947^722^, (2) represent one of only two SST proxies available across the entirety of the Phanerozoic, and (3) are commonly measured on foraminifera from drill cores. However, the *δ*^18^O_*carbonate*_ data are commonly affected by diagenesis, with ~30% of the 81,633 entries flagged as altered. Despite their limited temporal availability, ${{\rm{U}}}_{37}^{K{\prime} }$ data account for a quarter of all entries, and all data, barring those from regions prone to sea ice, are considered to reflect a primary SST signal. The high volume of ${{\rm{U}}}_{37}^{K{\prime} }$ data is likely a result of the ease of analysis and straightforward relationship between temperature and proxy value. The remaining three proxies collectively contribute to the remaining quarter of the entries in PhanSST, with conodont *δ*^18^O_*phosphate*_ being the least common.

**Table 4:** Summary of entries by proxy. See text and caption of Fig. 5 for details regarding how preservation was assessed.

Proxy	All entries		Unaltered entries	
	N	Proportion	N	Proportion
*δ*^18^O_carbonate_	81,633	54%	56,691	48%
δ^18^O_phosphate_	6,358	4%	6,014	5%
Mg/Ca	13,249	9%	9,846	8%
TEX_86_	14,091	9%	11,176	9%
${{\rm{U}}}_{86}^{K{\prime} }$	35,360	24%	35,324	30%
Total	150,691		119,051	

Below, we highlight some of the first-order spatial and temporal trends in data density, sampling locations, and proxy values of PhanSST. The syntheses presented illustrate just a small fraction of the spatio-temporal patterns uniquely elucidated by a compilation of this size and demonstrate the potential of the database to facilitate paleoclimatic, geochemical, and paleoecological research.

#### Temporal trends in data density

**Fig. 4:**
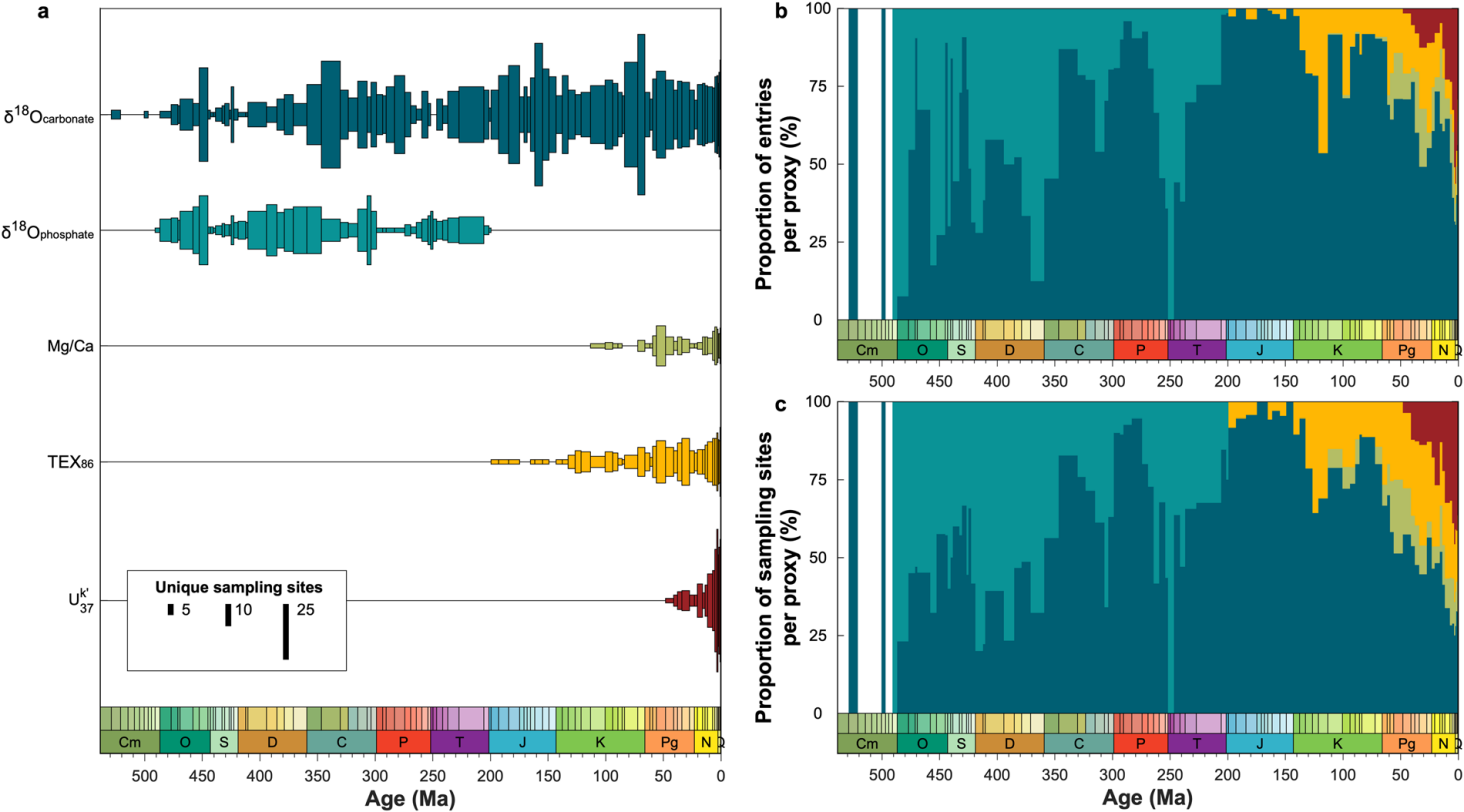
Summary of the (**a**) number of unique sampling locations through geologic time, (**b**) proportion of entries, and (**c**) proportion of unique sampling sites separated by proxy type and binned by geologic stage. Colors in panels **b** and **c** follow the convention of panel **a**.

**Fig. 5:**
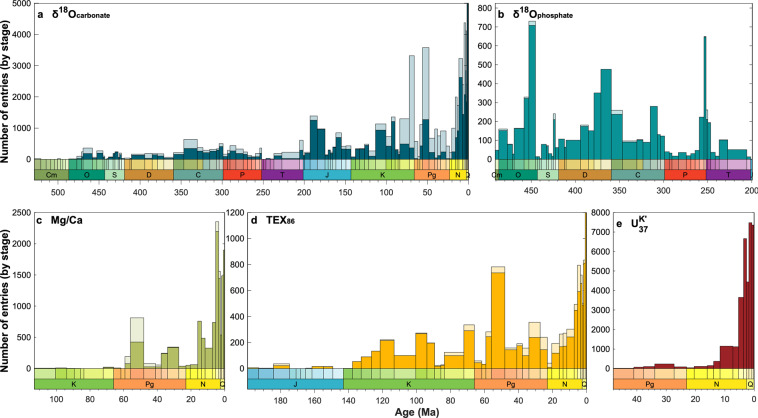
Number of entries from unaltered (opaque) and total (transparent) material, separated by proxy type and binned by geologic stage. The diagenetic determinations in panels **a-c** are based on the expert-assigned *DiagenesisFlag* field. This flag applies to all proxies barring TEX_86_ and ${{\rm{U}}}_{37}^{K{\prime} }$; here, TEX_86_ fidelity is based on entries with BIT, MI, and ΔRI values all below 0.5, while ${{\rm{U}}}_{37}^{K{\prime} }$ data whose paleolatitude is greater than 70°N are considered compromised by alkenone production in sea ice. Due to a large increase in the number of entries approaching the Holocene, the y-axis has been scaled in panels **a** and **d**, cropping the upper limits of the Quaternary data from these plots.

The Cenozoic accounts for just 12% of Phanerozoic time, while the Mesozoic accounts for 35% and the Paleozoic, 53%. The number of PhanSST entries and sampling sites per era, however, follow a different distribution (Table 5), reflecting fundamental differences in the availability, density, and nature of paleo-SST archives across geologic time. Cenozoic entries account for 79% of the data in PhanSST, while Cenozoic sampling sites account for a quarter of the unique sites. The over-representation of Cenozoic entries reflects the fact that the majority of these data come from drill cores, with long and often high-resolution records, and many of these cores have SST data from multiple proxies. Additionally, several of the proxies, such as ${{\rm{U}}}_{37}^{K{\prime} }$^723^, are restricted to more recent times. Collectively, these factors influence both the number of entries and unique sampling sites from each proxy type through time (Figs. 4,5). While carbonate oxygen isotope data, largely from planktonic foraminifera, dominate the Cenozoic record, many of these data have been flagged as altered due to pervasive secondary bottom water calcite precipitation overprinting the SST signal^466,724^. TEX_86_ data are common throughout the Cenozoic, while both the number of entries and sampling sites of the ${{\rm{U}}}_{37}^{K{\prime} }$ data grow rapidly across the late Neogene through Holocene. Overall, despite rising numbers of entries across the era, the number of unique sampling sites at the stage level remains fairly consistent within each proxy, with only the ${{\rm{U}}}_{37}^{K{\prime} }$ sites showing a large increase towards the present (Fig. 4). The abundance of multi-proxy records from the same cores further moderates the total number of unique sites across all proxies.

**Table 5:** Summary of the proportion of entries and sites by geologic era, with the proportion of Phanerozoic time encompassed within each era for comparison.

Era	Proportion of...		
	Phanerozoic	Entries	Sites
Cenozoic	12%	79%	24%
Mesozoic	35%	12%	35%
Paleozoic	53%	9%	41%

Mesozoic entries account for just 12% of the compilation but contribute over a third of all sampling sites (Table 5). The Mesozoic marks a transition in both depositional environment and proxy availability. In the Cretaceous, IODP data are still available, as are foraminiferal-based and TEX_86_ records. However, the the lack of pelagic calcifiers and preserved biomarkers in sediments predating the mid to late Mesozoic limits both the materials and proxies available. Further, nearly all seafloor older than the late Jurassic has been subducted^701^, which fundamentally restricts the temporal reach of ocean drill cores. Therefore, records from the early portion of the Mesozoic dominantly come from shelf deposits or epeiric seas. These data are generally collected from outcrops and thus lack the high-resolution, high sampling density of core data. Additionally, the early Mesozoic marks the initial breakup of Pangaea; the supercontinent reduced continental margin area and prohibited widespread continental flooding^725,726^ and so compared to other intervals in Earth’s history, there are very few marine environments preserved^727^. The reduction in early Mesozoic marine sedimentary occurrences is mirrored in PhanSST by the dearth of Triassic and early Jurassic sampling sites (Fig. 4b). Overall, the era is dominated by carbonate isotope data. Phosphate isotope data are common in the Triassic, but disappear at the close of the period, reflecting the extinction of conodonts^728^. Conversely, TEX_86_ data make an appearance in the latter half of the Mesozoic, and foraminiferal Mg/Ca data, though present in the Cretaceous, are infrequent, both in terms of entries and sampling sites.

Despite comprising more than half of the Phanerozoic Eon, Paleozoic entries account for just 9% of all data but constitute 40% of sampling sites (Table 5). All Paleozoic entries come from marine sediments deposited on continental margins or interiors. Paleozoic studies often report multiple sample sites, with only a small number of measurements from each site^67,252,616^. Thus, the number of sampling sites remains high, but the data density at each site is drastically reduced (Fig. 5). Only carbonate and phosphate oxygen isotope data are available for the Paleozoic (Fig. 4). Notable declines in data density in the Cambrian, Silurian, and Permian (Figs. 4 and 5) can be ascribed to a combination of preservation and eustasy, limiting the number of sites and material available for analysis.

#### Spatial trends in data density

**Fig. 6:**
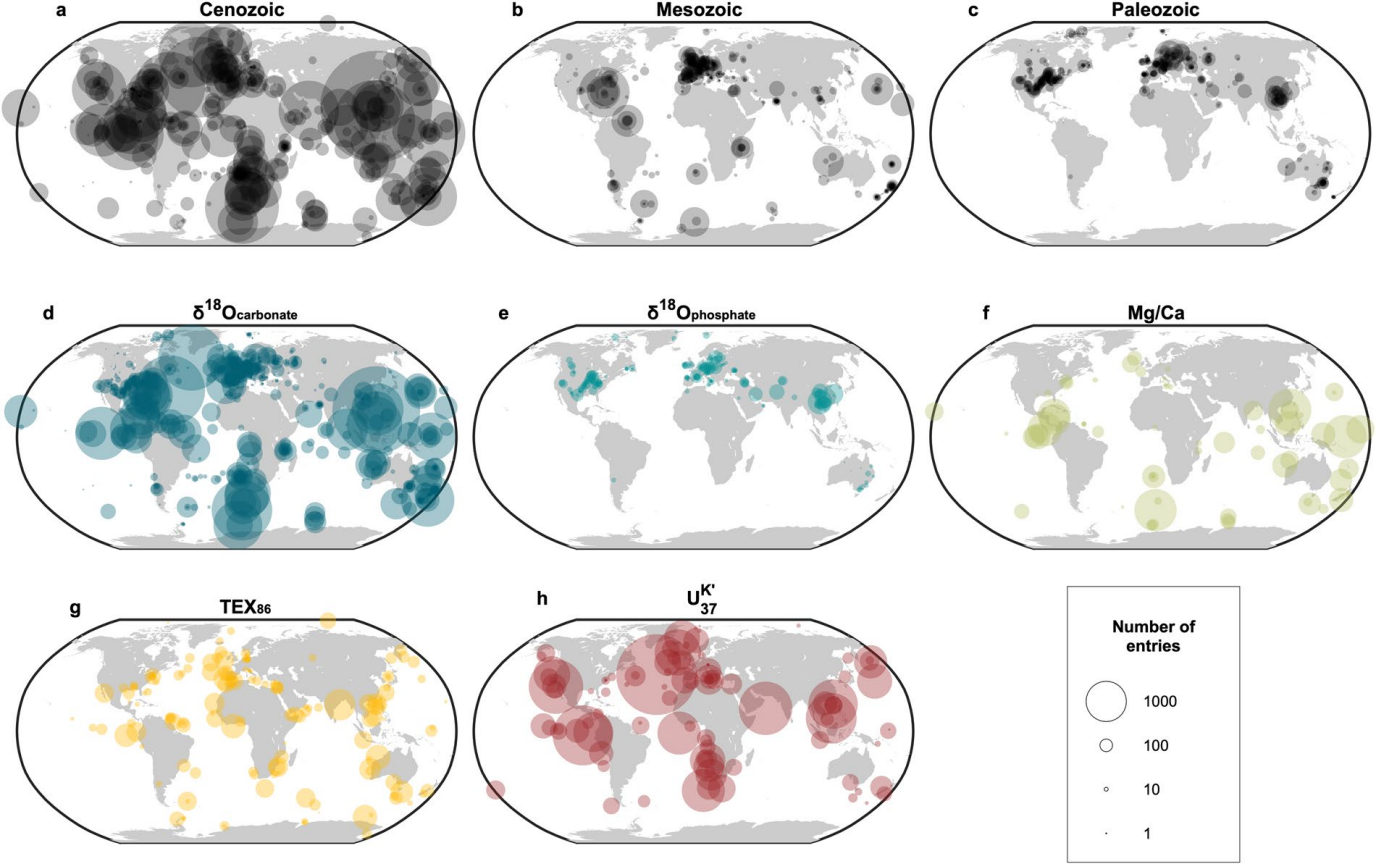
Summary of the modern spatial distribution of sampling sites by (**a**–**c**) era and (**d**–**h**) proxy type, with the size of each point scaled to the number of entries at each site. All panels are plotted on the same scale.

**Fig. 7:**
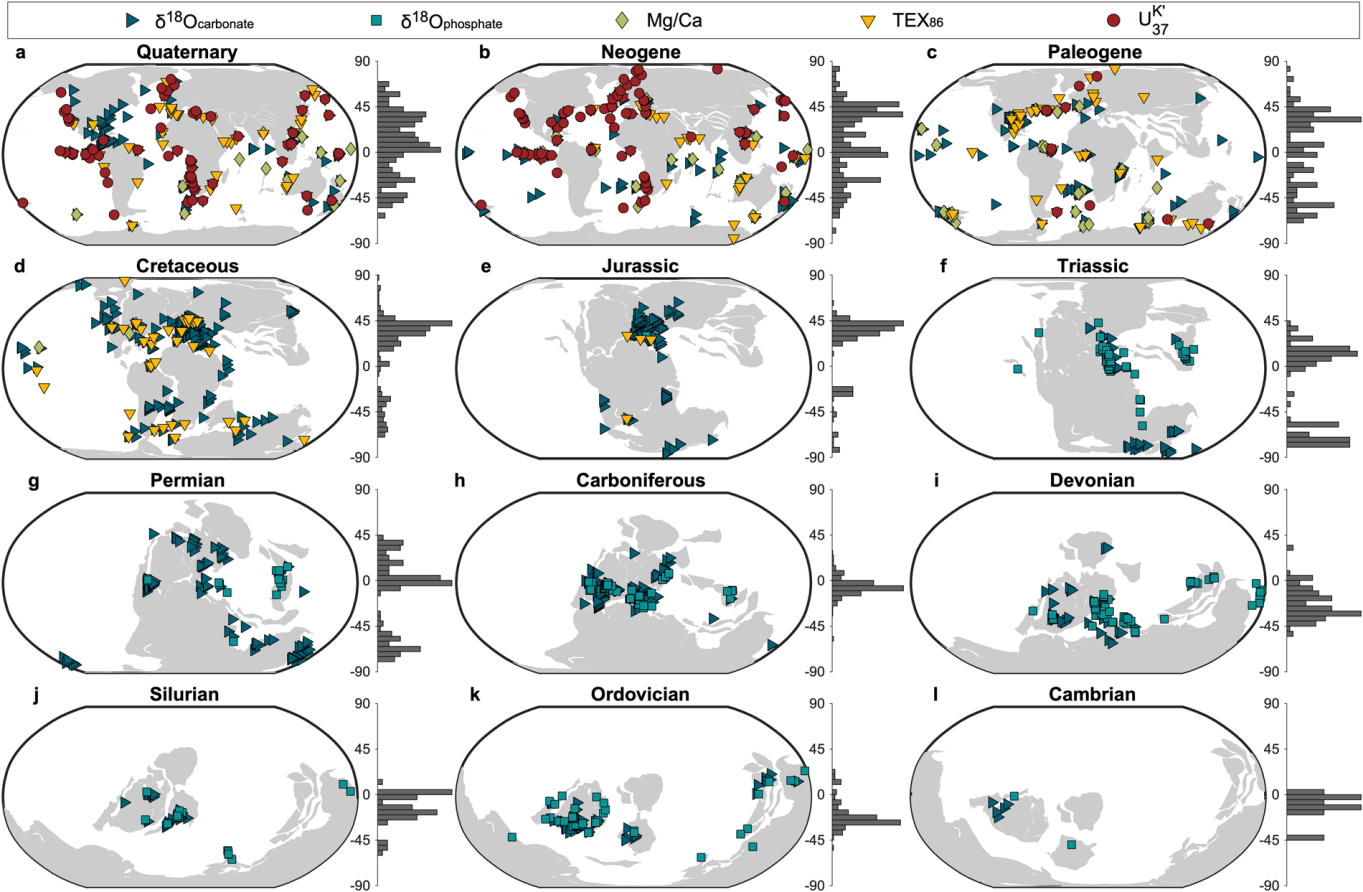
Summary of the paleogeographic spatial distribution of sampling sites, colored by proxy type and separated by geologic period. Histograms to the right of each map show the relative latitudinal distribution of all unique sampling sites within 5° bins.

The spatial distribution of PhanSST sampling sites are inherently uneven, both with respect to their modern (Fig. 6) and paleo-locations (Fig. 7). Paleo-latitude and -longitude of each entry was estimated using the plate model of Scotese and Wright^707^, implemented in G-Plates (Version 2.2.0)^729^. In terms of their modern distributions, Cenozoic data are the most spatially widespread and entry numbers at each site are high, owing to the availability of ocean core data and the high-resolution, multi-proxy studies they permit. Mesozoic sampling sites reflect the mix of high sample densities at the few ocean cores that extend back into the Cretaceous and lower sample density associated with outcrop data. By the Paleozoic, all samples are situated on land and heavily weighted toward the Northern Hemisphere, with most data coming from a few key regions (i.e., North America, Europe, Australia, and China). The Southern Hemisphere is consistently underrepresented across all three eras and five proxies and spanning both marine and continental deposits. The paucity of data from South America and Africa, as well as the southern sectors of the Indian and Pacific oceans, mirrors patterns in paleontological data^709,730^ and highlights the tectonic, depositional, and colonial biases in paleoclimate data.

Viewing the paleogeographic distribution of the data by period (Fig. 7) further accentuates depositional and latitudinal biases. The Cambrian through Jurassic data overwhelmingly come from epeiric sea settings, while the Cretaceous through Quaternary records are biased toward continental margins. Likewise, the Paleozoic records, which dominantly come from modern-day North America and Europe, are weighted toward the tropics; as these continents migrate northward through the Mesozoic and into the Cenozoic, the records begin to favor the Northern Hemisphere mid latitudes. The Cenozoic data are more evenly distributed across latitudes due to the availability of core data; however, the Southern Hemisphere remains underrepresented.

#### Spatio-temporal trends in proxy values

**Fig. 8:**
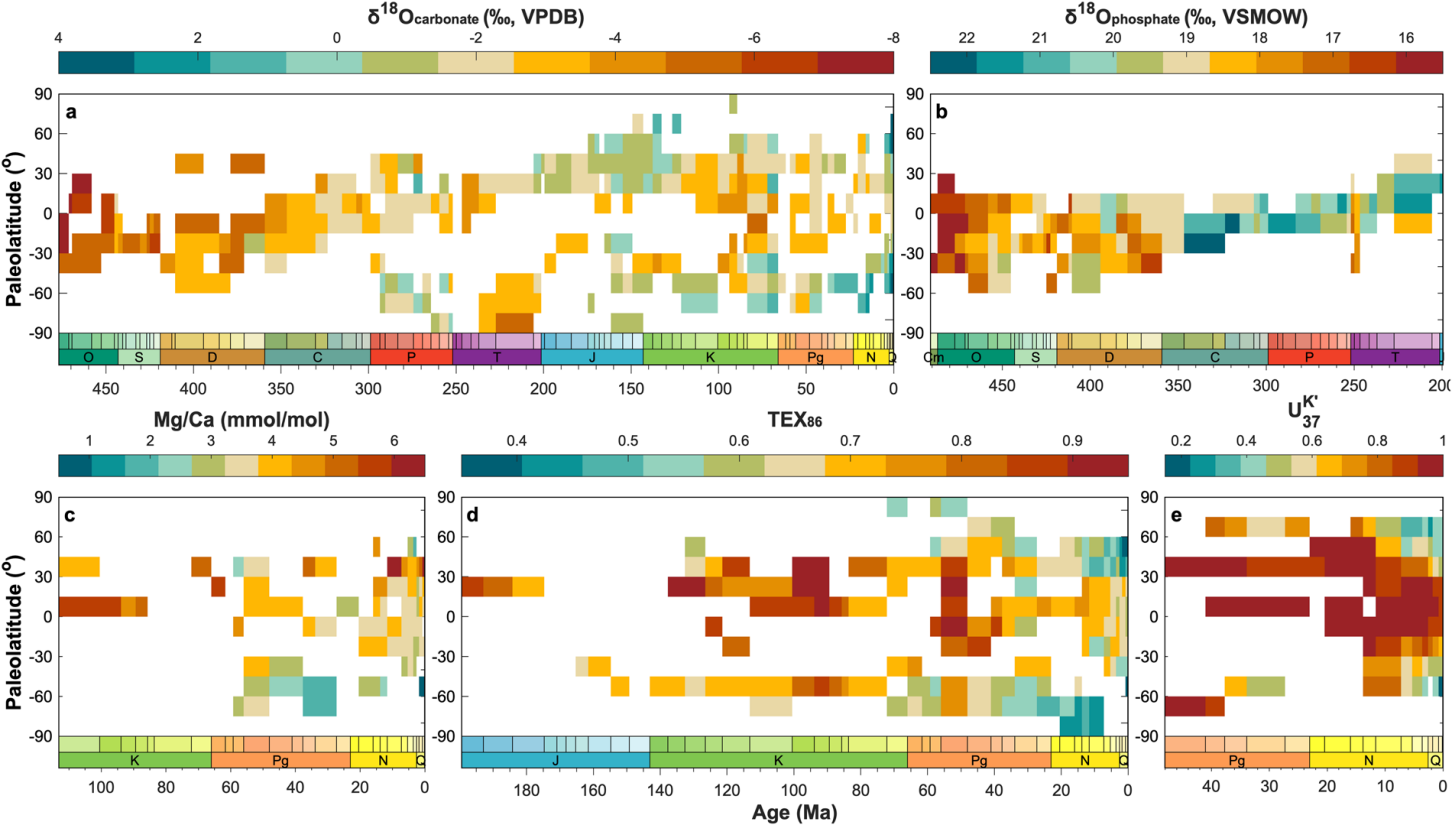
Summary of the spatio-temporal trends in proxy values from unaltered materials, separated by proxy type and binned temporally by stage and spatially by 15° paleolatitudinal bins. The scale of each color bar is unique to each proxy type, but for all panels, cooler colors correspond with proxy values associated with cooler temperatures and vice versa.

The volume of data contained within PhanSST and the consistent and queryable metadata fields permit a large-scale view of the evolution of proxy information through geological time. Figure 8 shows heat maps of proxy values from unaltered material across the temporal range of each proxy, with data temporally averaged by stage and spatially averaged into 15° paleolatitudinal bins. Vertical trends show the latitudinal proxy gradient at any given stage, while the horizontal trends show temporal evolution of proxy values within a latitudinal bin.

The *δ*^18^O_*carbonate*_ data (Fig. 8a) exhibit a clear trend toward higher values towards modern day. This trend has long been recognized and interpreted to reflect either a secular evolution in the oxygen isotopic evolution of seawater through geologic time, a true temperature signal from a much warmer ancient world, or an increased influence of diagenetic alteration with age^24,312,731,732^. Viewing the data as a function of both space and time importantly highlights that very few early Paleozoic records extend beyond the tropics, while tropical Mesozoic and (unaltered) Cenozoic data are less common. Thus, to some extent the observed shift toward higher values should logically follow based on the differences in the latitudinal extent of the data, regardless of the prevailing climate regime. The end of the Paleozoic also marks a transition from data sourced from warmer epeiric environments to continental margins, where temperatures are more likely to resemble zonal mean values^39^. Differences in the geographic and environmental spread of data between eras may therefore partly explain the long-term temporal trend.

The *δ*^18^O_*phosphate*_ data are restricted mainly to the low latitudes in the Paleozoic, boreal mid latitudes in the Mesozoic, and absent in the Cenozoic (Fig. 8b). While there is a long-term temporal trend toward higher values across the Paleozoic, the enrichment observed in the tropics during the Carboniferous and Permian is consistent with cooling during the Late Paleozoic Icehouse^733^.

The Mg/Ca data have a sporadic temporal and spatial distribution (Fig. 8c). Even during more data dense intervals, such as the late Neogene, latitudinal gradients deviate from a temperature-driven expectation. The Mg/Ca proxy is sensitive to several non-thermal factors, such as the magnesium to calcium ratio of the seawater at the time of precipitation, the bottom water calcite saturation state at the time of burial, surface ocean pH and salinity, and the cleaning method used during analysis^35–37^. Cumulatively, these factors inhibit straightforward interpretation of the raw proxy values.

Compared to the other data types, ${{\rm{U}}}_{37}^{K{\prime} }$ data have a short temporal span but a wide spatial distribution. Nearly all latitudinal bins from the mid-Neogene through modern are represented (Fig. 8e). Unencumbered by non-thermal influences, the alkenone data exhibit well developed latitudinal gradients. However, because ${{\rm{U}}}_{37}^{K{\prime} }$ is a ratio bounded on a 0 (cold) to 1 (hot) scale, tropical locations consistently approach the limit of the proxy, making it challenging to discern temporal trends in these regions. Though lacking the spatial coverage of the ${{\rm{U}}}_{37}^{K{\prime} }$ data, the TEX_86_ data exhibit spatial and temporal trends broadly consistent with the current understanding of Cenozoic climate^3^ and have the most consistent high latitude southern hemisphere coverage (Fig. 8d).

The paleogeographic maps (Fig. 7) and heatmaps (Fig. 8) demonstrate that all proxies have discontinuous spatial and temporal coverage, but that collectively the Cenozoic is well represented. Geographic coverage diminishes in the Mesozoic and is extremely limited in the Paleozoic. Differences in the distribution of data, both between eras and proxies, likely bias our collective understanding of Phanerozoic climate. Identifying and acknowledge these patterns can aid in the interpretation of global climate trends and inform decisions regarding where to target future data acquisition efforts, with respect to both geography and proxy type.

## Usage Notes

### Informed user notice

Although PhanSST has many applications beyond its initial intended purpose, several sources of uncertainty are not constrained in the current version of the compilation. As noted above, the database does not include age uncertainty. Additionally, while specific screening procedures (e.g., XRD, SEM) can help identify altered material, the assessment of preservation is subjective, particularly in a binary sense. We have taken a conservative approach, flagging everything that was either (1) interpreted as altered in the original publication, (2) was deemed suspect based on the expert opinion of the co-authors or (3) was beyond the reasonable range of values for any given proxy. As discussed above, since there are a wide range of metrics and thresholds used to evaluate the fidelity of different proxy values, we have chosen to include proxy-specific fields that can aid in diagenetic assessments. We recommend that end-users utilize these fields, in tandem with the binary *DiagenesisFlag*, to make their own informed diagenetic determinations.

All entries in PhanSST contain raw proxy values. This decision was intentional to ensure consistency between PhanSST and the original data tables from which the data were drawn and to ensure traceability. However, where appropriate, the database includes relevant information to assist in species- or methodological-specific corrections (e.g., *AnalyticalTechnique, NBS120c, Durango, CleaningMethod*). We encourage users to make use of these fields to guarantee appropriate reuse.

Finally, we would like to recognize that the data contained within PhanSST represent the collective work of countless researchers and required an enormous amount of time, effort, and resources to generate. PhanSST has compiled these disparate data sets, but can stake no claim in their generation. Upon reuse (and when realistically feasible), we recommend users to cite both PhanSST and the original data references to ensure appropriate attribution.

### Applying the database in deep time

When applying these data in the deep time, it will often be necessary to (1) convert the proxy values to temperature estimates and (2) estimate their paleogeographic position. In addition to the considerations outline above in the *Informed user notice*, it is important to make deliberate and justifiable decisions regarding the choice of temperature calibration and plate rotation model.

For any given proxy system, there are a variety of different PSMs available with which to estimate SST. There is justification to use different calibrations under different circumstances (e.g., based on the location, age, or taxon from which the data derive) and in many instances it may also be useful to report estimates of SSTs using multiple calibrations (see the *Which metadata fields are excluded and why?* section for specific examples). Similarly, in order to calculate SST, many of these proxies require assumptions about the seawater chemistry of ancient oceans and other non-thermal variables. In Table 6 we have provided a non-exhaustive list of calibration references and potential non-thermal predictor variables for each proxy system. Some of the PSMs are straightforward transfer functions^366,704,734^, allowing SSTs to be calculated by hand or, e.g., in Excel, while others involve a system of equations^31,34,35^ that can be implemented in various coding languages (e.g., Python, R, or Matlab). It is important to be aware that different PSMs also handle uncertainty differently, with some providing 1*σ* calibration uncertainty, some providing 95% confidence intervals, and others providing no means of estimating error.

**Table 6:** Non-exhaustive list of non-thermal predictor variables associated with each proxy system in PhanSST, and some commonly-used calibrations.

Proxy system	Non-thermal assumptions	Calibrations
*δ*^18^O_*carbonate*_	*δ*^18^O of the seawater	Bemis *et al*.^734^
	pH of the seawater	Grossman and Ku, 1986^735^
		Kim and O’Niel, 1997^736^
		Malevich *et al*.^34^
		See also Grossman, 2012^737^ for more
*δ*^18^O_*phosphate*_	*δ*^18^O of the seawater	Lécuyer *et al*.^366^
		Pucéat *et al*.^738^
Mg/Ca	Mg/Ca of the seawater	Anand *et al*.^739^
	pH of the seawater	Gray and Evans, 2019^36^
	Bottom water saturation state (Ω)	Tierney *et al*.^35^
	Salinity	
	Cleaning method (oxidative or reductive)	
TEX_86_		Schouten *et al*.^740^
		Kim *et al*.^704^
		O’Brien *et al*.^696^
		Tierney and Tingley, 2014^31^
${{\rm{U}}}_{37}^{K{\prime} }$		Müller *et al*.^741^
		Conte *et al*.^742^
		Tierney and Tingley, 2018^33^

Note that in addition to the listed non-thermal assumptions, some calibrations may also take into account other factors, such as the paleolatitude or the ocean basin from which the data derive.

There are, similarly, myriad plate rotation models to choose from and the choice of which model is most appropriate depends upon several factors. The most important consideration is how the paleogeographic information will be used. If the data will be plotted on top of an ESM output, then to ensure the data are placed correctly the user will want to use the same rotation model as the simulation. This is particularly true in deeper time, when paleolongitudes are unconstrained^38^. Once the user has determined the appropriate plate model, rotation of the data can be implemented using G-Plates^729^, either through the command line in Python or via a graphical user interface. Conversely, if the data are being used to investigate latitudinal temperature gradients during a specific time slice, then it may be prudent to try several rotation models to get a sense of the uncertainty in the values. If only paleolatitude is needed, we recommend using the Paleolatitude Calculator^38^, which provides estimates–and uncertainties–from several different plate rotation models.

### Data availability and nature of a living database

A static copy of PhanSST Version 0.0.1 is archived in the NOAA-NCEI Paleoclimatology Database (https://www.ncei.noaa.gov/access/paleo-search/study/36813)^26^. Version controlled releases of the database and additional reference and database metadata can be found on Zenodo (10.5281/zenodo.7049233)^27^ and at the PhanSST website (https://www.paleo-temperature.org). We chose to host the data on Zenodo because it (1) permits version control and (2) interfaces directly with GitHub, which will allow us to release new versions of the database under the same DOI. Despite our best efforts to identify data from the literature and QC each entry, given the sheer volume of data contained within PhanSST, there are undoubtedly errors or data sets that we have overlooked. Any issues or omissions identified by end-users can be reported on the PhanSST website and the erroneous information will be updated in future releases of the database. Likewise, the website contains a blank data entry template and instructions for entering and submitting missing or newly published data. Completed data entry forms can be submitted via the PhanSST website or emailed directly to PhanSST@outlook.com. We encourage the community to contribute newly published data so that the database can continue to grow. Through continued crowd-sourcing of data entry and QC, PhanSST will remain a useful resource for the paleoclimate community for years to come.

## Data Availability

Figures 4–8 were produced in Matlab. Example code and auxiliary functions to (1) reproduce Figs. 4–6 and (2) run the automated QC checks on the database are available on GitHub (https://github.com/EJJudd/SciDataSupplement). The paleocoordinates used to produce Figs. 7,8 were estimated using the plate model of Scotese and Wright^707^, implemented in G-Plates (Version 2.2.0)^729^.
